# Genome-Wide Genomic and Functional Association Study for Workability and Calving Traits in Holstein Cattle

**DOI:** 10.3390/ani12091127

**Published:** 2022-04-27

**Authors:** Michalina Jakimowicz, Joanna Szyda, Andrzej Zarnecki, Wojciech Jagusiak, Małgorzata Morek-Kopeć, Barbara Kosińska-Selbi, Tomasz Suchocki

**Affiliations:** 1 Biostatistics Group, Department of Genetics, Wrocław University of Environmental and Life Sciences, Kożuchowska 7, 51-631 Wrocław, Poland; michalina.jakimowicz@upwr.edu.pl (M.J.); joanna.szyda@upwr.edu.pl (J.S.); barbara.kosinska@upwr.edu.pl (B.K.-S.); 2 National Research Institute of Animal Production, Krakowska 1, 32-083 Balice, Poland; andrzej.zarnecki@iz.edu.pl (A.Z.); wojciech.jagusiak@urk.edu.pl (W.J.); malgorzata.morek-kopec@urk.edu.pl (M.M.-K.); 3 Faculty of Animal Science, University of Agriculture in Krakow, al. Mickiewicza 24/28, 30-059 Kraków, Poland

**Keywords:** calving traits, gene ontology, metabolic pathways, mixed model

## Abstract

**Simple Summary:**

Traits related to the calving abilities and workability of cows are essential both for the dairy economy and for the welfare of cows. We analysed the direct and maternal components of calving ease, direct and maternal components of stillbirth, milking speed, and temperament by looking for SNPs, gene ontologies, and metabolic pathways significantly associated with the additive genetic variability of those traits. At the genetic level, 150 significant SNPs were identified whereas at the functional level we found 45 significant gene ontology terms and 5 metabolic pathways.

**Abstract:**

The goal of our study was to identify the SNPs, metabolic pathways (KEGG), and gene ontology (GO) terms significantly associated with calving and workability traits in dairy cattle. We analysed direct (DCE) and maternal (MCE) calving ease, direct (DSB) and maternal (MSB) stillbirth, milking speed (MSP), and temperament (TEM) based on a Holstein-Friesian dairy cattle population consisting of 35,203 individuals. The number of animals, depending on the trait, ranged from 22,301 bulls for TEM to 30,603 for DCE. We estimated the SNP effects (based on 46,216 polymorphisms from Illumina BovineSNP50 BeadChip Version 2) using a multi-SNP mixed model. The SNP positions were mapped to genes and the GO terms/KEGG pathways of the corresponding genes were assigned. The estimation of the GO term/KEGG pathway effects was based on a mixed model using the SNP effects as dependent variables. The number of significant SNPs comprised 59 for DCE, 25 for DSB and MSP, 17 for MCE and MSB, and 7 for TEM. Significant KEGG pathways were found for MSB (2), TEM (2), and MSP (1) and 11 GO terms were significant for MSP, 10 for DCE, 8 for DSB and TEM, 5 for MCE, and 3 for MSB. From the perspective of a better understanding of the genomic background of the phenotypes, traits with low heritabilities suggest that the focus should be moved from single genes to the metabolic pathways or gene ontologies significant for the phenotype.

## 1. Introduction

Traits related to the calving abilities and workability of cows are essential both for dairy economics and for the welfare of cows. Difficult calvings and stillbirths entail veterinary costs, increased labour, and subsequent problems with fertility and may result in decreased yields of production traits [1,2]. The behaviour of cows during milking as well as milking speed influence the labour related to the milking process [3]. According to Philipsson et al. [4], in 1994 only five countries, the Nordic countries and Slovenia, considered calving traits in their national selection indices. Nowadays, 20 countries carry out routine genetic evaluations of calving ease and 18 countries consider workability (www.interbull.org (accessed on 5 February 2022)). Calving abilities are low heritable traits. One of the recent estimates published for the Holstein-Friesian breed by Oliveira Junior et al. reported estimates for calving ease of 0.10 for heifers and 0.03 for cows and even lower estimates for calving survival (the equivalent of stillbirth) of 0.07 for heifers and 0.01 for cows [5]. These figures were in accordance with previously published estimates [1,6,7,8,9,10]. However, despite such low heritabilities, during the last decade it has been possible to conduct successful selections, resulting in an improvement in calving ease and a decrease in stillbirth rate, as demonstrated by Ma et al. [11]. It is, therefore, interesting to explore the genetic architecture of these traits by identifying the polymorphisms associated with calving traits and even possibly pinpointing the candidate genes. Numerous genome-wide association studies (GWAS) for calving performance have been conducted so far and were recently reviewed by Ma et al. [11].

On the other hand, traits related to handling cows on farms—summarised under the general term workability—have only recently been subjected to genetic evaluations, which are currently being carried out by 18 countries at an international level (www.interbull.org (accessed on 5 February 2022)). Similar to calving traits, both components of workability—milking speed and temperament—have a relatively low heritability, but their additive genetic components are higher than those for calving traits, oscillating at around 10% of the total phenotypic variance [5,7,12,13]. So far, only three GWAS results for workability traits have been published by Jardim et al. [14], Marete et al. [15], and Chen et al. [16], indicating that further studies on various populations are needed for a better understanding of the genetic components of workability phenotypes.

Therefore, the primary goal of our study was to identify the SNPs significantly associated with the direct and maternal components of calving ease, direct and maternal components of stillbirth, cow temperament, and milking speed. However, having in mind the low heritabilities of all those traits and then, consequently, a lack of genes with very strong individual effects, we defined a secondary goal of the study, which was the identification of the metabolic pathways and gene ontology terms significantly associated with workability and calving. To achieve these goals, we first used a mixed model with random residual polygenic and additive SNP effects. The use of the residual polygenic effect allowed us to account for the incomplete linkage disequilibrium. Secondly, we detected the genes that were not more than 5000 bp from the SNP. Next, based on the SNP effects and information on the metabolic pathways and gene ontologies assigned to the genes, we implemented a mixed model for the calculation of the association between the metabolic pathways and the given trait.

## 2. Materials and Methods

The analysed data set originated from the Holstein-Friesian dairy cattle population. For each bull, two workability and four calving traits in the form of de-regressed breeding values (DRP) corresponding with the Interbull genomic evaluation from April 2020 were available. The phenotypic data came from the national conventional evaluation and MACE evaluation presented by Interbull. The analysed traits comprised direct calving ease (DCE), maternal calving ease (MCE), direct stillbirth (DSB), maternal stillbirth (MSB), milking speed (MSP), and temperament (TEM). The total number of bulls used in the analysis was 35,203. Detailed information about the number of individuals for each trait together with the heritabilities are summarised in Table 1.

The majority of individuals (87%) was genotyped by Illumina BovineSNP50 BeadChip Version 2, which contained 54,609 SNPs. Bulls genotyped with other platforms (Illumina BovineSNP50 BeadChip Version 1, 12% and Illumina EuroG10K BeadChip, 1%) were imputed into the above using Beagle software [17]. The SNP preselection criteria comprised a minor allele frequency of at least 0.01 and a technical quality of genotyping expressed by a minimum call rate of 99%. After editing, 46,216 SNPs remained for a further analysis.

The following mixed model was used to estimate the additive effects of the SNPs$:\;y=Xb+Z_{1}g+Z_{2}a+e\;$ where y represents a vector of the DRPs for the considered traits; *b* is a vector of the fixed effects representing a general mean with a design matrix X; $Z_{1}$ is a design matrix for the SNP genotypes, which is parameterised as −1, 0, or 1 for a homozygous, heterozygous, or an alternative homozygous SNP genotype, respectively; g is a vector of the random additive SNP effects; $Z_{2}$ is a design matrix for a residual polygenic effect; a is a vector of the random residual additive polygenic effects of the bulls representing the part of the additive polygenic variance that is not explained by the SNPs; and e is a vector of the residuals with $e\sim N\left(0,D{\hat{\sigma }}_{e}^{2}\right)$ where D is a diagonal matrix containing the reciprocal of the effective daughter contribution per bull on the diagonal and ${\hat{\sigma }}_{e}^{2}$ represents the residual variance. The covariance structure of g was assumed to be $g\sim N\left(0,I\frac{{\hat{\sigma }}_{a}^{2}}{N_{snp}}\right)$ with I being an identity matrix, ${\hat{\sigma }}_{a}^{2}$ representing the additive genetic variance of a given trait, and *N_SNP_* being the number of SNPs. This led to $a\sim N\left(0,A{\hat{\sigma }}_{a*}^{2}\right)$ where A is the numerator relationship matrix and ${\hat{\sigma }}_{a*}^{2}$ is a predetermined ratio of the additive polygenic variance for each trait that was set to ${\hat{\sigma }}_{a*}^{2}=0.4\cdot {\hat{\sigma }}_{a}^{2}$, i.e., a value that provided the best model fit. 

The estimation of the parameters of the above model was based on solving the mixed model equations proposed by Henderson [18]: $\left[\begin{array}{c} \hat{b}\\ \hat{g}\\ \hat{a} \end{array}\right]={\left[\begin{array}{ccc} X^{T}R^{-1}X & X^{T}R^{-1}Z_{1} & X^{T}R^{-1}Z_{2}\\ Z_{1}^{T}R^{-1}X & Z_{1}^{T}R^{-1}Z_{1}+G_{1}^{-1} & Z_{1}^{T}R^{-1}Z_{2}\\ Z_{2}^{T}R^{-1}X & Z_{2}^{T}R^{-1}Z_{1} & Z_{2}^{T}R^{-1}Z_{2}+G_{2}^{-1} \end{array}\right]}^{-1}\left[\begin{array}{c} X^{T}R^{-1}y\\ Z_{1}^{T}R^{-1}y\\ Z_{2}^{T}R^{-1}y \end{array}\right]$ where $R=D{\hat{\sigma }}_{e}^{2}$, $G_{1}=I\frac{{\hat{\sigma }}_{a}^{2}}{N_{snp}}$**,** and $G_{2}=A{\hat{\sigma }}_{a*}^{2}$. Consequently, the variance of y is then given by $Z_{1}G_{1}Z_{1}^{T}+Z_{2}G_{2}Z_{2}^{T}+R$.

For testing the hypotheses ($H_{0}:g=0$ vs. $H_{1}:g\neq 0$), we used the Wald test: $W=\frac{\hat{g}}{\sigma_{\hat{g}}}$ where $\sigma_{\hat{g}}$ is a standard error of the estimated SNP effect $\hat{g}$. Under *H_0_*, this statistic followed the standard normal distribution_._ The multiple testing correction was carried out via the Bonferroni approach [19]. The average of the lambda coefficient was 1.027 with the minimum value equal to 1.018 for DCE and the maximum value was 1.038 for TEM. 

To associate the SNPs with the closest genes, all polymorphism coordinates from the Illumina BovineSNP50 BeadChip were remapped to the ARS_UCD1.2 *Bos taurus* reference genome using biomaRt [20] implemented in Bioconductor and the NCBI Remap tool. The genomic annotation of the SNPs was then undertaken by using Variant Effect Predictor software [21] with the maximum distance between an SNP and its closest gene set to 5000 bp. The functional information was expressed by the pathways defined by the Kyoto Encyclopedia of Genes and Genomes (KEEG) and by gene ontology (GO) terms corresponding with the genes marked by the SNPs from the Illumina BovineSNP50 BeadChip. DAVID software [22] was used for the annotation. The estimation of the KEGG pathways and GO terms was carried out based on the following mixed model: $y^{*}=u+Z^{*}p+e^{*}\;$ where $y^{*}$ is the absolute value of the SNP effect for a given trait, p is a random KEGG pathway or GO term effect, and $e^{*}$ represents the residual term. $Z^{*}$ is the incidence matrix KEGG pathway or GO term effect p. It was assumed that $p\sim N\left(0,P{\hat{\sigma }}_{p}^{2}\right)$ and $e^{*}\sim N\left(0,L{\hat{\sigma }}_{e^{*}}^{2}\right)$ where P is a covariance matrix between the KEGG pathways or GO terms consisting of the percentage of common genes between them and ${\hat{\sigma }}_{p}^{2}$ represents the KEGG pathway or GO term variance. L is a diagonal matrix of the dimension N_SNP_ × N_SNP_ contained on the diagonal 1 (if an SNP was assigned to a KEGG pathway or a GO term) or 10 (if an SNP was not assigned any functional element); ${\hat{\sigma }}_{e^{*}}^{2}$ is the residual variance. For testing the hypotheses $H_{0}:p=0$ vs. $H_{1}:p\neq 0$, the Wald test was used as defined above. Similar to Model (1), the Bonferroni multiple testing correction was used to modify the nominal *p*-values [19]. 

## 3. Results

### 3.1. Direct Calving Ease

**Figure 1:**
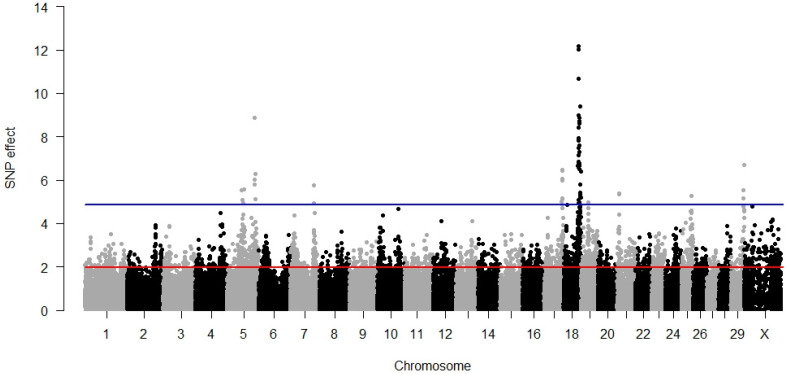
GWAS results for direct calving ease. The red line (1.96) represents a nominal and the blue line (4.88) a Bonferroni-corrected critical value corresponding with a 5% significance level.

The largest number of significant SNPs was detected for DCE amongst all analysed traits. The GWAS results, visualised in Figure 1, demonstrated an accumulation of 22 highly significant SNPs on chromosome 18 distributed across a region spanning 10,225,739 bp located close to the end of the chromosome. The average distance between the significant SNPs on BTA18 was 328,863 bp with a minimum equal to 29,504 bp and a maximum equal to 1,614,805 bp. Due to this, it was necessary consider several independent significant regions on BTA18. Significant SNPs were also identified on other chromosomes: BTA5, BTA7, BTA17, BTA19, BTA21, BTA25, and BTA29. All the significant SNP explained 12.45% of the phenotypic variance. The summary of significant SNP associations with DCE is presented in Supplementary Table S1. In this table, it can be seen that the majority (except 3 within-gene doublets and 15 intergenic SNPs) of SNPs significant for direct calving ease marked a different gene; as a result, 41 genes could be considered as candidates.

By moving the scope from the genes to the functions classified through GO terms, we identified seven significant biological processes: cartilage condensation, cell aggregation, artery development, nucleus localisation, cardiac myofibril assembly, myofibril assembly, and actin filament-based movement. Microfilament motor activity was the significant molecular function whereas the two significant cell components were the myosin complex and neuromuscular junction (Table 2). No metabolic pathway specific for the manifestation of a direct calving ease phenotype was identified.

### 3.2. Maternal Calving Ease

**Figure 2:**
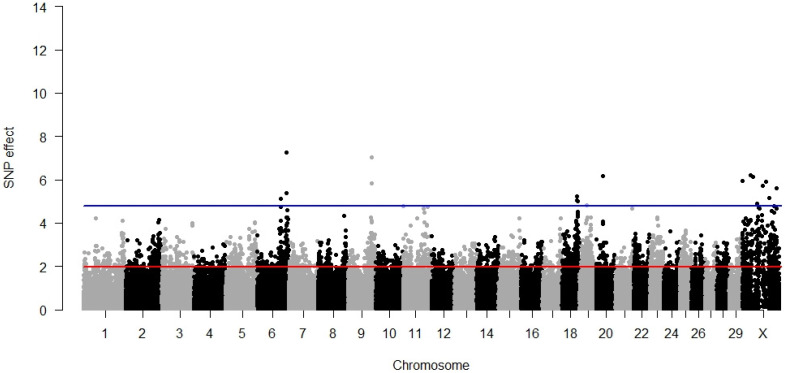
GWAS results for maternal calving ease. The red line (1.96) represents a nominal and the blue line (4.88) a Bonferroni-corrected critical value corresponding with a 5% significance level.

For the maternal component of calving ease, fewer significant SNPs (17) were identified than for the direct component (59). As is visible in Figure 2, the most significant SNPs (8) were scattered over almost the entire length of BTX from 863,652 bp up to 127,125,130 bp. All three SNPs from BTA18 were also significant for DCE, albeit with lower *p*-values. All the significant SNPs explained 3.58% of the phenotypic variance.

As for DCE, no significantly associated metabolic pathways were identified, but five gene ontologies revealed a significant association with MCE, which were different from the ontologies significant for the direct component (Table 2). These were processes of the positive regulation of protein maturation and the modulation of chemical synaptic transmission, with synapses being associated with cellular components. For the maternal component of calving ease, no significant molecular pathway was determined.

### 3.3. Direct Stillbirth

**Figure 3:**
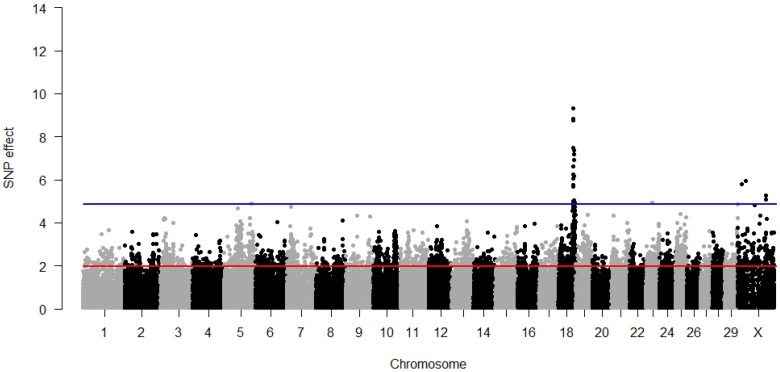
GWAS results for direct stillbirth. The red line (1.96) represents a nominal and the blue line (4.88) a Bonferroni-corrected critical value corresponding with a 5% significance level.

The GWAS results for direct stillbirth are visualised in Figure 3. Of the 25 significant SNPs, 19 overlapped with those that were also significant for DCE. Both traits were characterised by many significant SNPs located toward the end of BTA18; except for rs110508656, all the other 18 polymorphisms were pleiotropic for DSB and DCE. Another chromosome with multiple significant SNPs was BTX; however, each of the four polymorphisms pointed out at a very different location spanning as many as 192,213,034 bp. Moreover, single SNPs with a borderline significance were detected on BTA5 (*p* = 0.0468) and BTA23 (*p* = 0.0356). All the significant SNP explained 4.26% of the phenotypic variance. The significant GWAS results and their annotations are summarised in Supplementary Table S1. The similarity of the genetic architecture underlying DSB and DCE revealed by GWAS was further reflected on the functional annotation level because all eight GO terms significantly associated with direct stillbirth were also significant for DCE (Table 2). No significant association of stillbirth with the molecular pathways was determined.

### 3.4. Maternal Stillbirth

**Figure 4:**
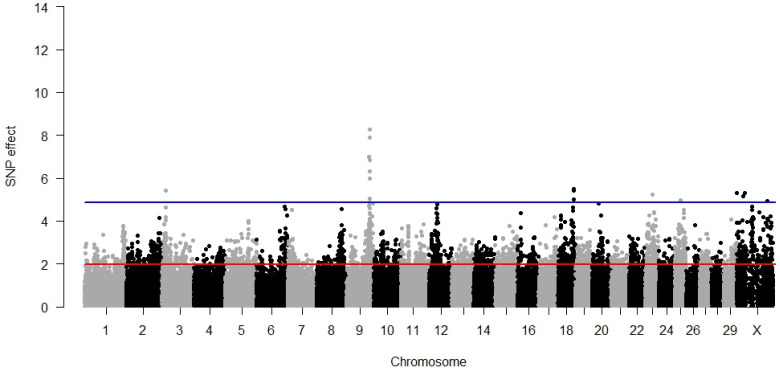
GWAS results for maternal stillbirth. The red line (1.96) represents a nominal and the blue line (4.88) a Bonferroni-corrected critical value corresponding with a 5% significance level.

The largest number (seven) of SNPs significant for MSB was located on BTA9. Except for rs43615598, they also exhibited the highest effects on MSB amongst all significant SNPs. The three SNPs located on BTA18 were also significant for the direct component of stillbirth (as well as for DCE); five SNPs located on BTA9, BTA18, and BTX were also associated with the maternal component of calving ease. All the significant SNP explained 10.82% of the phenotypic variance. The summary of SNPs significant for MSB is given in Supplementary Table S1 and a GWAS-based Manhattan plot is presented in Figure 4. Signalling mediated by cyclic nucleotides (in particular by cAMP) was the biological process associated with the maternal component of stillbirth whereas the significant molecular function comprised the binding of 1-phosphatidylinositol (Table 2). MSB was the only calving-related trait for which a significant KEGG metabolic pathway could be assigned (Table 3), linked to PD-L1 expression and the PD-1 checkpoint pathway in cancer (bta:05235) and Th1 and Th2 cell differentiation (bta:04658).

### 3.5. Temperament

**Figure 5:**
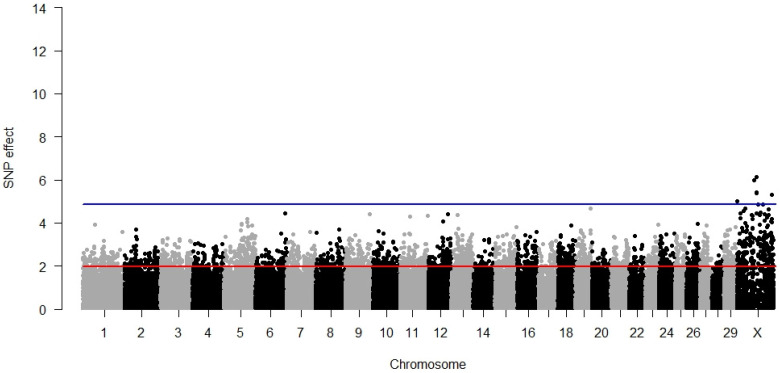
GWAS results for temperament. The red line (1.96) represents a nominal and the blue line (4.88) a Bonferroni-corrected critical value corresponding with a 5% significance level.

All seven SNPs significant for temperament were located on chromosome X, but across a very long span of 130,498,495 bp (Figure 5 and Supplementary Table S1). All the significant SNP explained 1.92% of the phenotypic variance. Eight gene ontology terms were estimated as significant for temperament, which were related to the processes of gamma-aminobutyric acid secretion and transport and the transmembrane import of calcium ions and glucose into cells as well as the early endosome membrane cell component (Table 2) The significant KEGG pathways were related to the metabolism of porphyrin, chlorophyll, glyoxylate, and dicarboxylate (Table 3).

### 3.6. Milking Speed

**Figure 6:**
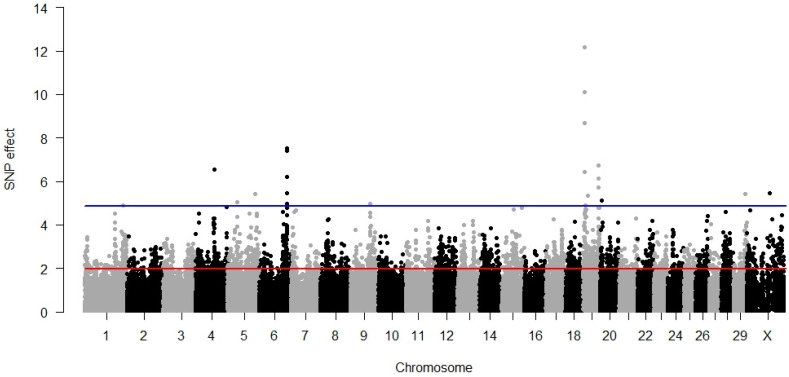
GWAS results for milking speed. The red line (1.96) represents a nominal and the blue line (4.88) a Bonferroni-corrected critical value corresponding with a 5% significance level.

With 1 exception, all 24 SNPs significantly associated with milking speed did not show pleiotropy with the other traits considered in this study. The most SNPs (ten) were located on BTA19 with the most significant of them forming two groups. One was close to the beginning of the chromosome, between 7,250,802 bp and 7,717,717 bp, and harboured two genes, ANKFN1 and a novel gene ENSBTAG00000038823. The other was close to the end of the chromosome, between 59,364,966 bp and 59,547,890 bp, and harboured a novel gene ENSBTAG00000048685. A group of five highly significant SNPs was also detected on BTA6; three of them were within a short region spanning 68,984 intergenic bps, close to the MSX1 gene. All the significant SNP explained 5.75% of the phenotypic variance. The summary and annotation of all significant SNPs are given in Supplementary Table S1 and the Manhattan plot of MSP is presented in Figure 6. Amongst the 11 gene ontology terms significantly influencing milking speed, there were the biological processes related to hippo signalling as well as the catalysis of ceramides and glycosphingolipids. The associated molecular functions comprised ligand-gated ion channel activity, phosphoric diester hydrolase activity, and voltage-gated calcium channel activity as well as the binding of calcium ions and 1-phosphatidylinositol (Table 2). The only significant KEGG pathway comprised the metabolism of the degradation of valine, leucine, and isoleucine (Table 3).

### 3.7. Pleiotropy

**Figure 7:**
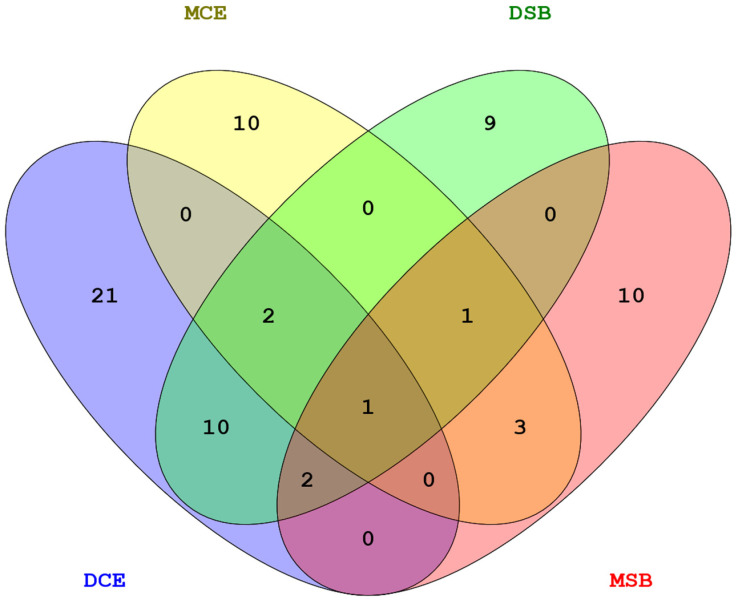
SNPs common amongst calving traits.

The number of SNPs common across the traits is visualised in Figure 7. No common SNPs with significant effects were identified between TEM and MSP, but amongst calving traits, we observed that a few overlapped, indicating a pleiotropic effect. A large number of 14 SNPs located on BTA18 were common between the direct components of calving ease and stillbirth. The maternal components of both traits shared six SNPs located on BTA9, BTA18, and BTX.

## 4. Discussion

Several genomic regions and GO terms as well as five KEGG pathways associated with the calving and workability traits were detected in our study.

As already indicated by Xiang et al. [23], fitting all variants simultaneously is the preferred method to identify variants causal for complex traits; an approach that was also used in our GWAS model. We also considered that, to capture missing heritability, rare variants should be incorporated into the analysis. Our investigation was carried out based on a standard Illumina 50K BeadChip microarray, which identified that many of the available SNPs were not placed directly into the genic sequence. As a result, the SNP gene and the following SNP metabolic pathway associations were mainly determined based on the linkage disequilibrium and not on a physical location. Moreover, the microarray was designed to contain SNPs with a relatively high MAF to provide a good description of the genomic variability in various cattle breeds and, therefore, deliberately did not include rare variants (www.illumina.com, accessed on 25 April 2022). Although the ideal data set would contain variants detected based on whole genome sequences, it is difficult to practically collect sequences of many individuals large enough to allow for the accurate statistical modelling of low heritable traits without a need for variant imputation, which itself provides an LD-based genotype approximation [23]. However, an indirect way to exploit the effects of all, common, and rare variants leads through the modelling of metabolic effects expressed by pathways or gene ontology terms. The importance of using metabolic information in the analysis of complex traits has been stressed in the literature during the last decade because it allows for the description of the biological basis of complex traits in a more intuitive, physiologically relevant way without a need to select particular genes from the metabolic system of an organism [24] Typical pathway-based analyses ignore the correlations between the pathways, which is a biological simplification because many genes are shared between pathways. Lee et al. [25] proposed an approach to estimate the pathway effects by incorporating the information on correlations between pathways; this is, however, statistically distinct from the approach proposed in our study.

Of the 22 significant SNPs detected for DCE on BTA18, 9 were also reported to be associated with direct calving ease: rs110389036, rs108984194, rs41582494, rs42843551, rs41664920, rs110889414, and rs110912084 by Müller et al. [26] and rs109478645 and rs109882115 by Abo-Ismail et al. [6]. For two other SNPs, significant associations with birth index and calf size—i.e., traits related to calving ease—were reported by Höglund et al. [27]. Furthermore, rs110312059 on chromosome 25 was previously reported to be associated with direct calving ease by Sahana et al. [28]. Moreover, mouse orthologues of four genes marked by significant SNPs were associated with disease phenotypes that could be regarded as analogous to calving ease: embryonic growth retardation as well as embryonic, foetal, and pre-weaning lethality.

Regarding MCE, amongst the significant SNPs on BTX, two had already been previously reported by Cole et al. [29] as associated with maternal calving ease (rs42766480) and direct calving ease (rs41567624). Amongst the other significant SNPs, an association with direct calving ease was reported for rs109399965 on BTA6 and rs41601571 on BTA20 by Sahana et al. [16]. It is noteworthy that many of the significant SNPs were reported to be associated with moderately to highly heritable conformation traits, which may then indirectly impact calving ease in a maternal inheritance mode, i.e., body depth (h^2^ = 0.21, 2 SNPs), dairy form (1 SNP), rump width (h^2^ = 0.30, 5 SNPs), stature (h^2^ = 0.54, 2 SNPs), and type (3 SNPs). A summary of the significant SNPs is given in Supplementary Table S1.

Five SNPs from BTA18 significant for DSB have already been reported for direct stillbirth by other studies: rs109478645 with the 2nd and rs109882115 with the 3rd overall highest effect by Höglund et al. [27]; and rs41664920, rs110889414, and rs110508656 by Müller et al. [12]. The last polymorphism was located within the tweety family member 1 gene (TTYH1) whose mouse orthologue was associated with embryonic lethality and embryonic growth arrest disorders. One of the four significant SNPs located on BTX, rs110344484, was also reported to be associated with DSB by Cole et al. [29].

Different from the previously described traits, in the case of the GWAS results for MSB, no significant SNP has been reported in the literature as being associated with the trait. However, a significant SNP on BTA25 was located within the calcineurin-like EF-hand protein 2 gene (CHP2), which is associated with pre-weaning lethality in mice. Both KEGG pathways significant for MSB are related to the immune response through T-type lymphocytes.

Regarding the workability traits, the ATPase copper transporting alpha gene (ATP7A) harbouring rs41623769, which was one of the seven SNPs with the highest effect for TEM, is associated with temperament-related phenotypes of lethargy and hypoactivity in mice. One of the SNPs significant for MSP on BTA19 (rs29020026) was reported to be associated with several udder conformation traits such as front and rear teat placement, udder attachment, udder cleft, and udder depth and height by Cole et al. [29]; rs41257416 from BTA5 was previously reported by Jardim et al. [14] as being associated with milking speed. 

The pleiotropic effects of SNPs observed in our study were expected based on the high genetic correlations estimated for Norwegian Red cattle by Heringstad et al. [30] between the direct components of stillbirth and calving difficulty as well as between the maternal components of these traits; these amounted to 0.79 and 0.62, respectively.

## 5. Conclusions

Calving traits are of low heritability. Their additive genetic component did not typically exceed 10% (see our results in Table 1 and a recent review from Ma et al. [2]). Therefore, the presence of single genes with marked effects was not anticipated for the traits. All the significant markers depended on the trait explained from a 3.58% (MCE) to a 12.45% (DCE) phenotypic variance. However, a surprisingly large overlap was observed between the genomic regions; therefore, many of the polymorphisms detected as being significant in our study have already been indicated by previous analyses (listed in the Results and Discussion sections). This observation is important for carrying out the genomic selection of the traits, especially by incorporating targeted polymorphisms into the evaluation model. Another situation was investigated for workability traits; the lack of common significant SNPs between the workability traits suggested no common genetic background of the phenotypes from this group of traits. From the basic perspective of a better understanding of the genomic background of the phenotypes, the low heritabilities of the traits indicated that the focus should be moved from single genes to the metabolic pathways or gene ontologies significant for the manifestation of calving problems or workability. Such a functional-based approach has so far not been considered either in relation to calving or the workability traits. 

## Figures and Tables

**Table 1:** Number of bulls and heritability.

Trait	Number of Bulls	Heritability
DCE	30,603	0.05
MCE	29,738	0.04
DSB	24,521	0.03
MSB	28,081	0.05
TEM	22,301	0.09
MSP	28,376	0.12

DCE: direct calving ease; MCE: maternal calving ease; DSB: direct stillbirth; MSB: maternal stillbirth; TEM: temperament; MSP: milking speed.

**Table 2:** Significant GO terms.

Trait	GO Term	Effect	*p*-Value
DCE	GO: 0001502	4.8154	2.30 × 10^−3^
	GO: 0098743	4.8154	2.30 × 10^−3^
	GO: 0060840	8.6306	3.05 × 10^−18^
	GO: 0000146	8.6306	3.05 × 10^−18^
	GO: 0051647	8.6306	3.05 × 10^−18^
	GO: 0055003	8.6306	3.05 × 10^−18^
	GO: 0030239	8.6306	3.05 × 10^−18^
	GO: 0016459	8.6306	3.05 × 10^−18^
	GO: 0030048	8.6306	3.05 × 10^−18^
	GO: 0031594	8.6306	3.05 × 10^−18^
MCE	GO: 1903319	4.1611	4.92 × 10^−2^
	GO: 0099572	4.5622	7.90 × 10^−3^
	GO: 0050804	4.5622	7.90 × 10^−3^
	GO: 0060076	4.5622	7.90 × 10^−3^
	GO: 0014069	4.5622	7.90 × 10^−3^
DSB	GO: 0060840	4.6114	6.20 × 10^−3^
	GO: 0000146	4.6114	6.20 × 10^−3^
	GO: 0051647	4.6114	6.20 × 10^−3^
	GO: 0055003	4.6114	6.20 × 10^−3^
	GO: 0030239	4.6114	6.20 × 10^−3^
	GO: 0016459	4.6114	6.20 × 10^−3^
	GO: 0030048	4.6114	6.20 × 10^−3^
	GO: 0031594	4.6114	6.20 × 10^−3^
MSB	GO: 0005545	4.9310	1.30 × 10^−3^
	GO: 0019933	5.2822	2.00 × 10^−4^
	GO: 0019935	5.2822	2.00 × 10^−4^
TEM	GO: 0046717	5.5607	1.34 × 10^−8^
	GO: 0015812	5.5607	1.34 × 10^−8^
	GO: 0031901	5.5987	1.34 × 10^−8^
	GO: 1902656	8.5417	6.61 × 10^−18^
	GO: 0097553	8.5417	6.61 × 10^−18^
	GO: 0044381	4.4921	1.10 × 10^−2^
	GO: 2001275	4.4921	1.10 × 10^−2^
	GO: 2001273	4.4921	1.10 × 10^−2^
MSP	GO: 0022834	6.8714	3.17 × 10^−12^
	GO: 0015276	6.8714	3.17 × 10^−12^
	GO: 0016788	4.4982	1.07 × 10^−2^
	GO: 0008081	4.4982	1.07 × 10^−2^
	GO: 0005509	4.4982	1.07 × 10^−2^
	GO: 0035329	4.3080	2.56 × 10^−2^
	GO: 0005545	4.6258	5.80 × 10^−3^
	GO: 0005245	4.5538	8.20 × 10^−3^
	GO: 0046514	5.0346	7.00 × 10^−4^
	GO: 0046479	5.0346	7.00 × 10^−4^
	GO: 0030149	5.0346	7.00 × 10^−4^

**Table 3:** Significant KEGG pathways.

Trait	KEGG	Number of Significant SNPs	Number of Associated Genes	Effect	*p*-Value
MSB	bta: 05235	62	29	3.6479	3.20 × 10^−2^
	bta: 04658	62	29	3.6479	3.20 × 10^−2^
TEM	bta: 00630	34	15	3.7176	2.43 × 10^−2^
	bta: 00860	21	13	3.7114	2.49 × 10^−2^
MSP	bta: 00280	25	11	4.0403	6.50 × 10^−3^

## Data Availability

The data sets analysed for this study are available upon formal request to the EuroGenomics cooperative.

## Supplementary Materials

The following supporting information can be downloaded at: https://www.mdpi.com/article/10.3390/ani12091127/s1, Supplementary Table S1: SNPs significant in GWAS and their annotation.
